# Gastric mucosal proteomic analysis reveals the role of the TGF-*β* signaling pathway in gastritis associated with *Helicobacter pylori* infection

**DOI:** 10.3389/fmicb.2025.1565392

**Published:** 2025-09-16

**Authors:** Chengjie Zhu, Lijuan Xiong, Yun He, Chaoqin Sun, Fei Mo

**Affiliations:** ^1^Center for Clinical Laboratories, the Affiliated Hospital of Guizhou Medical University, Guiyang, China; ^2^School of Clinical Laboratory Science, Guizhou Medical University, Guiyang, China

**Keywords:** *Helicobacter pylori*-induced gastritis, proteomics, transforming growth factor beta 1, transforming growth factor-*β*-activated kinase, T helper cell 17, regulatory T cells

## Abstract

*Helicobacter pylori* (*H. pylori*) infection induces chronic inflammation in the gastric mucosa. There is a close relationship between Th17/Treg cell imbalance and adverse outcomes associated with *H. pylori* infection. The differentiation and development of both Th17 and Treg cells require the regulatory involvement of TGF-*β*. However, the regulatory role of the TGF-*β* signaling pathway in *H. pylori*-induced gastritis remains unclear. This study aimed to investigate the impact of *H. pylori* infection on the expression of TGF-β pathway proteins in patients with chronic gastritis, as well as the changes in Th17 and Treg cells and their cytokine expression levels. In this study, gastric mucosal proteomic analysis revealed that differentially expressed proteins were enriched in signaling pathways, such as TGF-*β*, mitogen-activated protein kinase (MAPK), and Th17. Experiments demonstrated that mRNA levels of TGF-β1 and TAK1, along with their protein expression, were increased in both the peripheral blood and gastric mucosa of *H. pylori*-infected gastritis patients. Compared to the negative control group, *H. pylori*-infected gastritis patients showed elevated levels of Th17 and Treg cell transcription factors (*RORγt* and forkhead box protein 3 (*Foxp3*) mRNA) in both peripheral blood and gastric mucosa. Additionally, there was an increase in IL-17 and Foxp3 protein expression in the gastric mucosa along with elevated levels of IL-17 and IL-10 in peripheral blood. Animal experiments revealed a decreased ratio of Th17/Treg in *H. pylori*-infected gastritis mice. We hypothesize that *H. pylori* infection may contribute to gastric mucosal inflammatory responses by activating the TGF-*β* signaling pathway and disrupting the immune balance between Th17 and Treg cells during inflammation. These findings provide new strategies for the prevention and treatment of *H. pylori*-induced gastritis.

## 1 Introduction

*Helicobacter pylori* (*H. pylori*) is a Gram-negative bacterium that colonizes the human gastric mucosa and persistently activates local inflammatory cascades (Camilo et al., 2017). Persistent *H. pylori*-induced chronic gastritis may progress to atrophic gastritis, peptic ulcers, and even gastric carcinoma (Duan et al., 2025). Epidemiological studies indicate that *H. pylori* infects approximately half of the global population, making it one of the most prevalent bacterial infections worldwide (Engelsberger et al., 2024). Failing to eradicate *H. pylori* represents a critical juncture in disease progression, highlighting the importance of controlling the advancement of *H. pylori*-associated chronic gastritis. Research has demonstrated that *H. pylori* infection activates multiple pro-inflammatory signaling pathways, contributing significantly to its persistent colonization and chronic infection of the gastric mucosa; however, the underlying mechanisms remain incompletely understood (Li et al., 2022).

Upon microbial infection, the host activates an immune response in which cellular immunity plays a pivotal role. In recent years, dysregulation of the balance between CD4^+^ T cell subsets—specifically Th17 and regulatory T (Treg) cells—has been recognized as a critical determinant influencing inflammatory outcomes (Thomas et al., 2023). Under the regulation of microenvironmental immune molecules, naïve T cells differentiate into Th17 and Treg cells. Th17 cells express the retinoic acid-related orphan receptor γt (RORγt) transcription factor and secrete cytokines such as IL-17 and IL-21, thereby promoting immune responses. These cells can amplify tissue damage via IL-17 while serving as essential effector cells in clearing pathogens (Kumar et al., 2021; Lee et al., 2020). Conversely, Treg cells express the forkhead box protein 3 (Foxp3) transcription factor and exert immunosuppressive functions in a Foxp3-dependent manner. By secreting cytokines such as IL-10, Treg cells induce and maintain immune tolerance. Although this suppresses excessive immune activation, it may also contribute to persistent chronic infections (Laragione et al., 2023; Tagkareli et al., 2022). Th17 and Treg cells exert opposing effects on inflammation, functionally antagonizing each other while collectively maintaining immune homeostasis (Liu et al., 2020). Studies indicate that persistent *H. pylori* infection alters the gastric mucosal microenvironment, leading to the dysregulation of T-cell subsets, with Th17 and Treg cells playing dominant roles in cell-mediated immunity (Nabavi-Rad et al., 2022). The Th17 response contributes to gastric inflammation against *H. pylori* infection. Upon antigenic stimulation, the host secretes multiple cytokines that drive inflammatory reactions. Conversely, an increase in Treg cells attenuates gastritis severity but concurrently suppresses bactericidal immunity, thereby facilitating bacterial persistence and chronic infection (Kedmi et al., 2022; Araújo et al., 2022). Therefore, within the *H. pylori*-colonized gastric microenvironment, Th17 and Treg cell responses do not operate as static, parallel pathways but rather engage in dynamic “mutual exclusion-complementarity” interactions, which together determine the intensity of inflammation and the outcome of bacterial colonization.

Current research indicates potential interactions between Th17 and Treg cells, with the differentiation of both cell subsets largely relying on TGF-*β* signaling (Smith et al., 2013; Hang et al., 2019). Among the members of the TGF-β family, TGF-β1 has been widely recognized as an immunomodulatory cytokine with “yin-yang” functionality. TGF-β1 synergizes with IL-6 to promote the differentiation of Th17 cells, which possess both immunoregulatory and pathogenic properties. At high concentrations, TGF-*β*1 induces Treg cell differentiation and enhances their immunosuppressive functions while simultaneously inhibiting pro-inflammatory cytotoxic T cells and Th cells. Consequently, the TGF-*β* signaling pathway plays a pivotal role in maintaining both immune tolerance and immune response (Abbas et al., 2013; Zou and Restifo, 2010; Aarts et al., 2022). In summary, while the TGF-β signaling pathway is crucial for restricting *H. pylori* colonization and modulating host inflammatory responses, its precise role in adult *H. pylori*-induced gastritis remains unclear, which has limited the development of intervention strategies for *H. pylori*-associated disease progression. Therefore, this study employed proteomic analysis of gastric mucosal biopsy specimens from *H. pylori*-infected gastritis patients to preliminarily investigate two key aspects: (Camilo et al., 2017) the potential regulatory role of the TGF-*β* signaling pathway during *H. pylori* infection and (Duan et al., 2025) its association with Th17/Treg cell dynamics. These findings may reveal novel therapeutic targets and provide important theoretical foundations for improving *H. pylori* eradication efficacy and preventing *H. pylori*-related diseases.

## 2 Materials and methods

### 2.1 Patient information

The study enrolled patients diagnosed with chronic gastritis through gastroduodenoscopy and histological examination at the Affiliated Hospital of Guizhou Medical University between June 2023 and March 2024. The inclusion criteria comprised the following: (1) no use of bismuth preparations, H2-receptor antagonists, proton pump inhibitors, antibiotics, or non-steroidal anti-inflammatory drugs (NSAIDs) within 4 weeks prior to endoscopy and (2) no history of gastric or duodenal surgery. Among 54 eligible patients, gastric mucosal biopsy specimens were evaluated using Warthin–Starry (W–S) staining, the rapid urease test (RUT), and the 14C urea breath test (14C-UBT) for group stratification. The patients were classified into the *H. pylori*-positive gastritis group (*n* = 24) if they had ≥2 positive test results or the *H. pylori*-negative gastritis group (*n* = 26) if all tests were negative. Cases with only one positive result (*n* = 4) were excluded. All participants provided informed consent, and the study protocol was approved by the Ethics Committee of the Affiliated Hospital of Guizhou Medical University.

### 2.2 Main reagents and instruments

The main reagents and instruments included the following: Gastric *H. pylori* detection kit (urease method) (Anxin Biotechnology Co., Ltd.); human peripheral blood lymphocyte isolate, HE staining kit, and spirochete silver staining kit, Warthin–Starry method (Beijing Solepol Science and Technology Co., Ltd.); TRIzol reagent (Thermo Fisher Scientific); reverse transcription reagent (Takara Bio); flow cytometer (BD FACSCantoTM Flow Cytometer); SYBR Fluorescence Quantification Kit (Novozymes Bioscience Co., Ltd.); Human TGF-β1 ELISA kit (Xinbosheng Bio-technology Co., Ltd.); Human IL-17 ELISA kit and Human IL- 10 ELISA kit (Jingmei Bioengineering Co., Ltd.); anti-TGF-β1 (ab215715, Abcm); anti-Foxp3 (ab215206, Abcam); anti-TAK1 (12330-2-P, Wuhan Three Eagles Biotechnology); p-TAK1 (AF3019, Affinity); enzyme labeling instrument (Thermo scientific); PCR detection system (Tianlong Technology Co., Ltd.); Transcription Factor Buffer Set (AB_2869424, BD Pharmingen); eBioscience™ Cell Stimulation Cocktail (00–4,975, Invitrogen™); PE Rat anti-Mouse Foxp3 (563,101, BD Pharmingen™); FITC anti-mouse CD4 Antibody (100,405, Biolegend); PE/Cyanine7 anti-mouse IL- 17A Antibody (506,921, Biolegend); and APC anti-mouse CD25 Antibody (102,011, Biolegend).

### 2.3 Specimen collection and preservation

Fasting venous whole blood samples (3 mL) were collected from the patients in the morning and preserved in EDTA-anticoagulated tubes. Following centrifugation, plasma and blood cells were aseptically separated into enzyme-free EP tubes under sterile conditions. The plasma samples were stored at −80 °C, while blood cells were subjected to lymphocyte isolation using Ficoll density gradient centrifugation, followed by preservation in TRIzol reagent at −80 °C. Gastric mucosal biopsy specimens were obtained from the lesser curvature of the gastric antrum by experienced endoscopists. A portion of each sample was fixed in 10% formalin for paraffin embedding, while the remainder was immediately stored at −80 °C for subsequent experiments.

### 2.4 Identification of *H. pylori*

The rapid urease test was performed by immediately placing the gastric mucosal biopsy specimens into urea enzyme reaction wells to observe color changes in the liquid medium. For hematoxylin and eosin (H&E) staining, the paraffin-embedded gastric mucosal sections underwent sequential processing, including routine dewaxing to hydration, nuclear staining with hematoxylin, cytoplasmic counterstaining with eosin, dehydration, and clearing, followed by mounting for microscopic examination. Warthin–Starry staining of the gastric mucosa involved processing the paraffin sections through standard dewaxing and hydration, followed by incubation with 1% silver nitrate at 60 °C for 45–60 min. The sections were then developed with a silver-gelatin solution at 60 °C for 2–5 min until a brown–black coloration appeared, followed by dehydration, clearing, and final mounting for microscopic evaluation.

### 2.5 Enzyme-linked immunosorbent assay

The plasma levels of the inflammatory factors TGF-β1, IL-10, and IL-17 were detected using their corresponding kits. The signals were detected using a spectrophotometer at 450 nm, and the ELISACalc software was used to analyze the results.

### 2.6 Real-time quantitative PCR

A total of 100 mg of gastric mucosa tissue was lysed in 1 mL of TRIzol reagent and used to extract the total RNA. The RNA was reverse transcribed with the TaqMan reverse transcription reagent to synthesize cDNA. RT-qPCR was performed using an Opticon RT-PCR system according to the manufacturer’s instructions. mRNA levels were calculated using the 2-ΔΔCt method, with GAPDH serving as the internal reference. The primer sequences are shown in Table 1.

**Table 1:** Sequence of the newly designed primers in the study.

Target gene	Primer name	Sequence (5’–3’)
*GAPDH*	Forward primer	TGGCGCTGAGTACGTCG
	Reverse primer	ACACCCATGACGAACATG
*RORγt*	Forward primer	CTGCAAGACTCATCGCCAAAG
	Reverse primer	TTTCCACATGCTGGCTACACA
*Foxp3*	Forward primer	ACAAGGGCTCCTGCTGCATCG
	Reverse primer	ATGAGCGTGGCGTAGGTGAAAGG
*TGF-β1*	Forward primer	CAGCAACAATTCCTGGCGATA
	Reverse primer	AAGGCGAAAGCCCTCAATTT
*TAK1*	Forward primer	CAGCCTCTGCCGCCTCCTC
	Reverse primer	GCTCCTCTTCCAACAACCTCTTCC

### 2.7 Immunohistochemistry

Immunohistochemical (IHC) staining was performed on 4-μm formalin-fixed, paraffin-embedded gastric mucosal sections to detect the expression of TGF-β1, TAK1, p-TAK1, Foxp3, and IL-17 proteins in the gastric mucosa. After deparaffinization, the sections were incubated with primary antibodies at 4 °C overnight, washed with PBS, and incubated with secondary antibodies for 30 min, followed by additional PBS washes. The sections were developed with a DAB solution, mounted, and examined microscopically. The ImageJ color deconvolution plugin was used to measure the area fraction (percentage of positive staining area) and integrated density for all sections, with the average optical density (AOD) calculated as the ratio of integrated density to area. Throughout the experiment, identical microscope parameters and illumination conditions were maintained. The AOD values between the groups were expressed as mean ± SD, and intergroup comparisons were performed using independent samples *t*-tests, with a *p*-value of < 0.05 considered statistically significant.

### 2.8 DIA proteomic analysis

Gastric mucosal biopsy samples from the lesser curvature of the gastric antrum were collected from six patients in both the *H. pylori*-positive gastritis group and the *H. pylori*-negative gastritis group, preserved at −80 °C, and transported under frozen conditions to the laboratory of Shanghai Majorbio Bio-pharm Technology Co., Ltd. The samples were thawed on ice and lysed with an appropriate volume of 8 M urea aqueous solution containing protease inhibitors, followed by 2 min of ultrasonication on ice and 30 min of ice-cold lysis. After centrifugation at 12,000 *g* for 30 min at 4 °C, the protein supernatant was collected for BCA protein quantification. Subsequent proteomic analysis was performed using an Orbitrap Astral high-resolution mass spectrometer. Based on protein expression quantification results, intergroup differential protein analysis was conducted using Student’s *t*-test, with screening thresholds set at |log_2_FC| ≥ 1 and a *p*-value of < 0.05. The identified differentially expressed proteins were then subjected to further biological analysis using Majorbio’s cloud bioinformatics platform.

### 2.9 Establish experimental animal models

The animal study was approved by the Animal Care and Ethics Committee of Guizhou Medical University. A total of 12 C57BL/6 mice (equal gender distribution) were randomly assigned to two groups: the model group (n = 6), which received 0.5 mL of *H. pylori* SS2000 strain suspension (1 × 10^9^ CFU/mL) via oral gavage every other day for five doses, and the control group (n = 6), which was administered an equivalent volume of sterile brain–heart infusion broth. All mice underwent 12-h fasting before and 4-h fasting after each gavage. Furthermore, 8 weeks after the final administration, the mice were euthanized for gastric mucosal collection. Model validation was performed through the urease test and HE staining to assess inflammatory changes, with successful model establishment defined by positive urease test results and histologically confirmed chronic gastritis.

### 2.10 Flow cytometry

Spleens from the *H. pylori-*infected mice were collected, prepared into single-cell suspension, and stimulated in 1640 complete medium at 37 °C for 5 h. Single-cell suspension of the spleens of the uninfected *H. pylori* mice served as the control group. The cells were stained with CD4 and CD25 antibodies for 30 min and fixed with fixation/permeabilization solution in the dark at 4 °C for 1 h. Finally, the cells were incubated with IL-17 and Foxp3 antibodies for 1 h at 4 °C, protected from light, and analyzed using a BD flow cytometer system and FlowJo V10 software.

### 2.11 Statistical methods

Data were analyzed using the SPSS 21.0 and GraphPad Prism 5.0 software. Data that followed a normal distribution were expressed as mean ± SD. Comparisons between the two groups were performed using the independent samples *t*-test, with a *p*-value of < 0.05 considered statistically significant.

## 3 Results

### 3.1 Pathologic features of the gastric mucosa in *H. pylori* chronic gastritis

**Figure 1:**
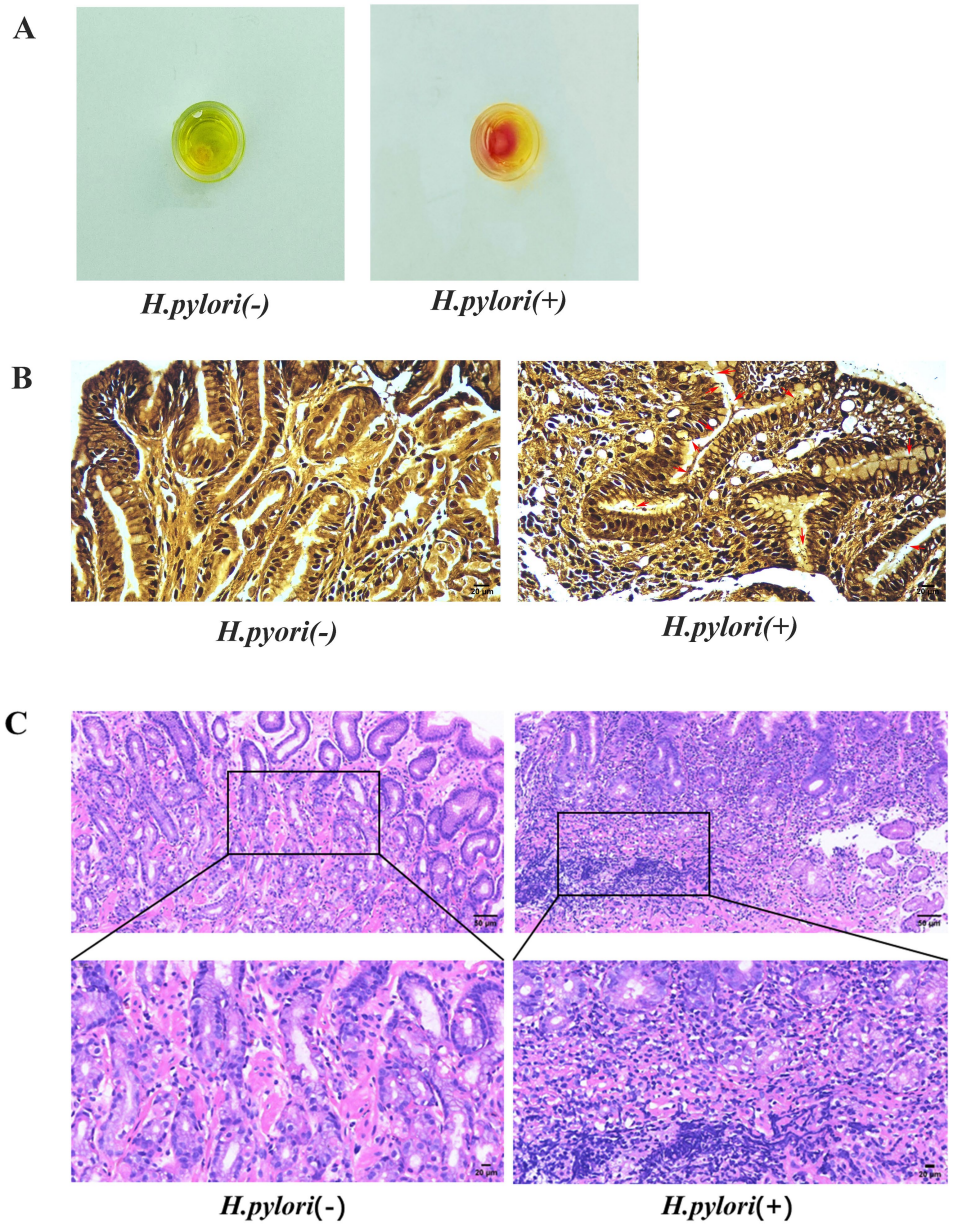
Identification of *H. pylori*. **(A)** Rapid urease assay of the gastric mucosa. **(B)** W–S staining of the gastric mucosa with *H. pylori* indicated by red arrows (W-S staining, magnification, 400×). **(C)** HE staining of the gastric mucosa (HE, magnification, 200×, 400×).

The rapid urease test revealed that the fluid surrounding the gastric mucosa turned red, indicating positive *H. pylori* infection. Areas without coloration were considered negative (Figure 1A). The W–S staining of the paraffin sections of the gastric mucosa showed black rods, short rods, or globules of *H. pylori* in the glandular lumen and margins of the gastric mucosa in the *H. pylori*+ group, and the background of the gastric mucosa in the negative group was clear (Figure 1B). The analysis of the HE staining revealed that the structure of the gastric mucosa was clear in the patients with *H. pylori-*negative gastritis, and there was mild inflammatory cell infiltration. The patients with *H. pylori*-negative gastritis exhibited normal gastric mucosal structure with minimal inflammatory cell infiltration, whereas those with *H. pylori-*positive gastritis showed extensive inflammatory cell infiltration, lymphoid tissue hyperplasia, and a reduced number of intrinsic glands throughout the mucosal layer (Figure 1C).

### 3.2 Proteomic analysis of the gastric mucosa in the patients with *H. pylori-*infected gastritis

#### 3.2.1 Identification of differentially expressed proteins in the gastric mucosa

**Figure 2:**
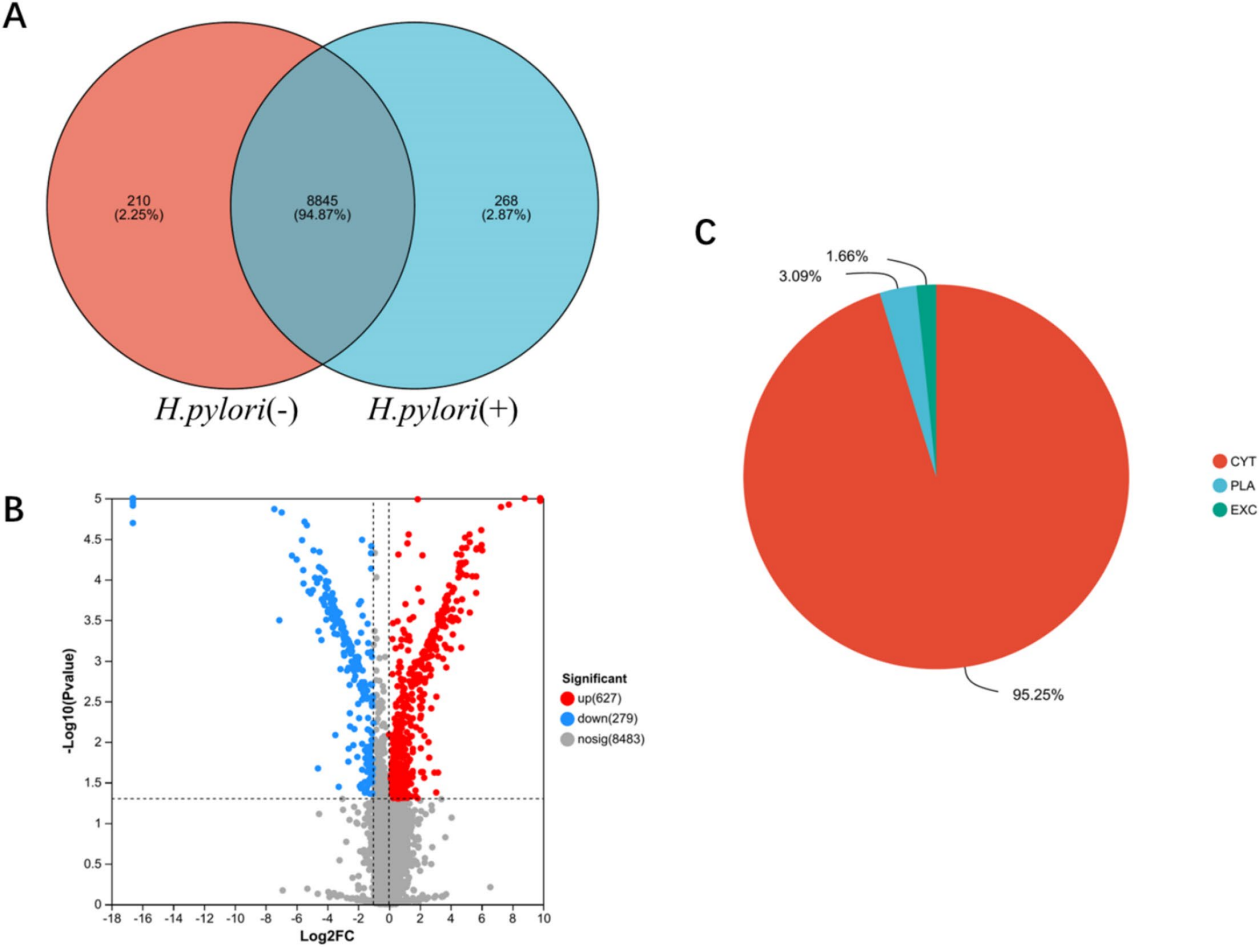
Identification of differentially expressed proteins in the gastric mucosa. **(A)** Venn analysis of gastric mucosal protein sets in the two groups. **(B)** Differential volcano plot of the gastric mucosa. **(C)** Subcellular localization pie chart of gastric mucosa differential proteins in the two groups. CYT (cytoplasmic), PLA (Plasma Membrane), and (EXC) Extracellular.

To better understand the potential pathogenic mechanisms of gastric mucosal inflammation following *H. pylori* infection, we conducted proteomic analysis of gastric mucosal tissues from both patient groups. The analysis of protein interactions between the two datasets revealed 268 unique proteins in the *H. pylori*-positive gastritis group and 210 unique proteins in the *H. pylori*-negative gastritis group (Figure 2A). Compared to the *H. pylori*-negative group, a total of 906 differentially expressed proteins were identified in the *H. pylori*-positive group, including 627 upregulated and 279 downregulated proteins, as demonstrated in the volcano plot of protein expression differences (Figure 2B). Subcellular localization analysis showed that these differentially expressed proteins were predominantly enriched in the cytoplasm (95.25%), followed by the plasma membrane (3.09%) and extracellular space (1.66%) (Figure 2C).

#### 3.2.2 Biological information analysis of differentially expressed proteins

**Figure 3:**
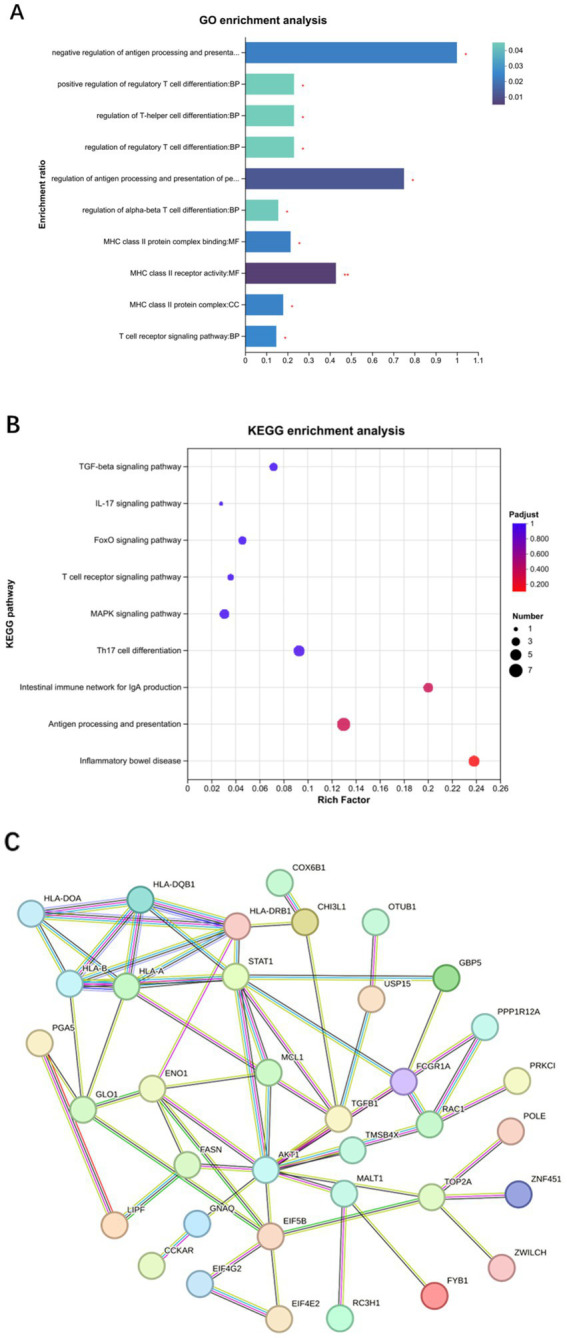
Biological information analysis of differentially expressed proteins. **(A)** GO enrichment analysis. **(B)** KEGG enrichment analysis. **(C)** Protein interaction network analysis.

The GO analysis of differentially expressed proteins was categorized into biological processes, cellular components, and molecular functions. The proteins were involved in biological processes, such as Th cell differentiation, positive regulation of cell differentiation, and T cell immune responses, while cellular components and molecular functions were associated with MHC class II protein complexes and receptor activity (Figure 3A). KEGG pathway analysis demonstrated significant enrichment of differentially expressed proteins in *H. pylori*-induced gastritis within pathways such as Th17 cell differentiation, the TGF-*β* signaling pathway, the mitogen-activated protein kinase (MAPK) signaling pathway, the IL-17 signaling pathway, and antigen processing and presentation (Figure 3B). Given the pivotal role of the TGF-β signaling pathway in T cell subset differentiation and inflammatory cytokine secretion following *H. pylori* infection, we analyzed 132 proteins associated with TGF-β signaling and T cell immune response processes, generating a network comprising 36 node proteins and 62 edges, with an average node degree of 3.44 and an average local clustering coefficient of 0.578. This analysis revealed complex interactions among differentially expressed proteins, with the network clearly demonstrating the TGF-*β*1 protein’s interactions with multiple proteins, including STAT1, which participates in regulating inflammatory cytokine secretion, and USP15, which plays a critical role in modulating various aspects of cellular immunity and inflammatory functions (Figure 3C). Furthermore, in the gastric mucosa of the patients with *H. pylori*-infected gastritis, USP15, ZNF451, TGFB1, and CCNT were identified as participants in the TGF-*β* signaling pathway, while FYB, HLA-DRB1, ANKRD24, and TMEM35 were implicated in T lymphocyte differentiation and immune response processes (Table 2). Bioinformatics analysis revealed that the host’s antibacterial immune response following *H. pylori* infection is primarily mediated by Th17 cells and their associated cytokines within the CD4 + T cell subset, with the TGF-*β* and MAPK signaling pathways potentially contributing to the regulatory mechanisms of T cell subset differentiation.

**Table 2:** List of 11 differential proteins that were significantly associated with *H. pylori*-infected gastritis.

Number	Accession	Symbol	Protein name	Regulate	FC
1	Q9Y4E8	USP15	Ubiquitin carboxyl-terminal hydrolase 15	Up	1.12
2	Q05DE9	FYB	FYB	Up	1.46
3	E9PH99	ZNF451	ZNF451	Up	2.20
4	A0A5K1TT74	HLA-DRB1	HLA	Up	5.24
5	A0A499FJK2	TGFB1	TGFB1	Up	31.43
6	P07996	THBS1	Thrombospondin-1	Up	1.29
7	A4L733	PDIA2	Protein disulfide-isomerase	Down	3.17
8	Q9UBU3	GHRL	Appetite-regulating hormone	Down	8.44
9	Q53FP2	TMEM35A	Novel acetylcholine receptor chaperone	Down	0.40
10	Q8N6D5	ANKRD29	Ankyrin repeat domain-containing protein 29	Down	0.34
11	O76076	CCN5	CCN family member 5	Down	0.47

### 3.3 Up-regulation of TGF-β1 and TAK1 expression during *H. pylori* infection

**Figure 4:**
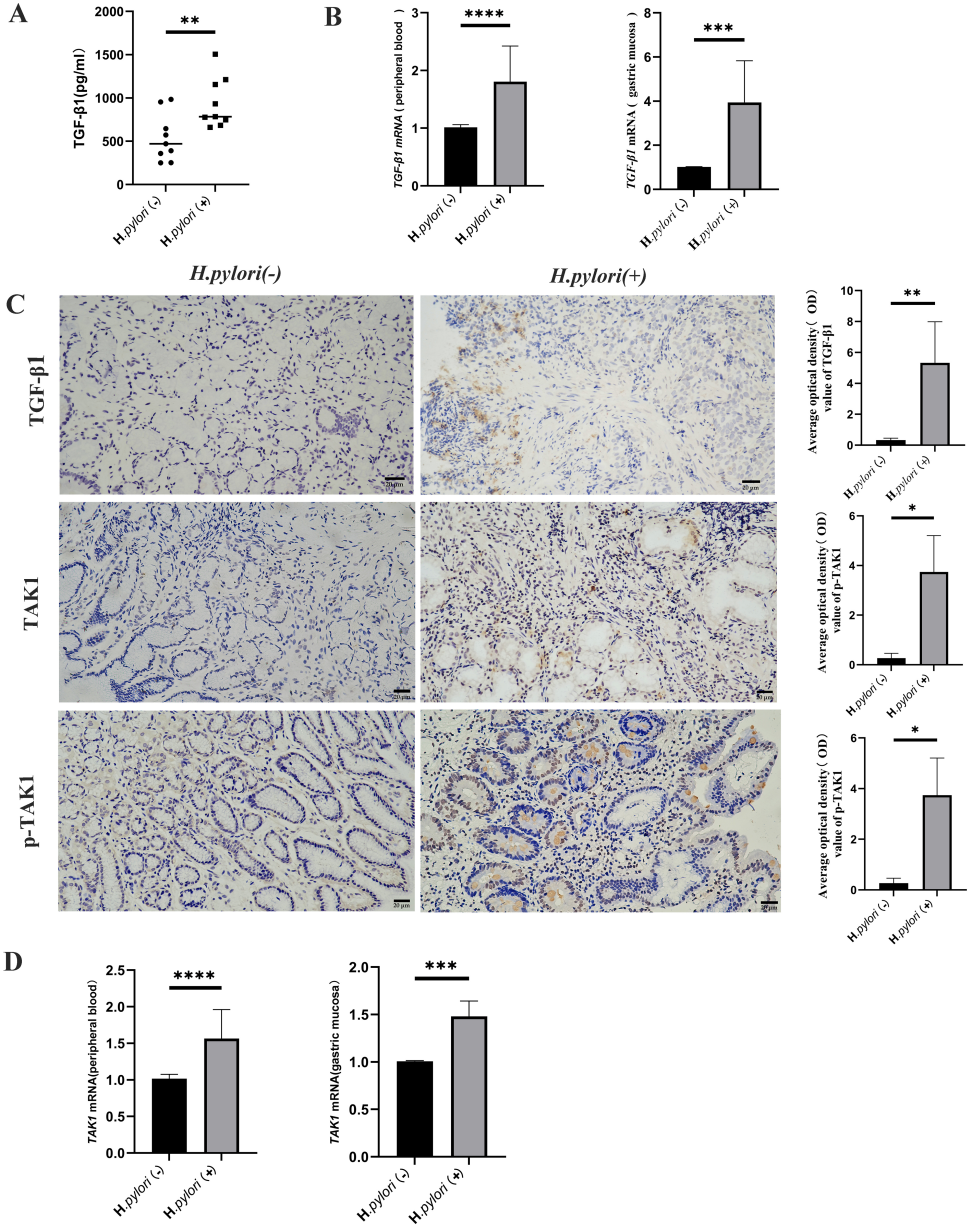
Up-regulation of TGF-β1 and TAK1 expression during *H. pylori* infection. **(A)** ELISA detection of the TGF-β1 expression level in the peripheral blood of the patients. **(B)** Real-time quantitative PCR to detect the TGF-β1 mRNA expression level in the peripheral blood and gastric mucosa of the patients. **(C)** IHC was performed to detect the expression of TGF-bβ1, TAK1, p-TAK1, and other proteins in the gastric mucosal tissue of the patients (IHC, magnification, 200×). **(D)** Real-time quantitative PCR analysis of TAK1 mRNA expression in the peripheral blood and gastric mucosa of the patients. n ≥ 3, data are presented as the mean ± SD. *P < 0.05, ****P < 0.001, ****p < 0.0001 (Student’s *t-*test).

Bioinformatics analysis revealed that differentially expressed proteins in the gastric mucosa of the patients with *H. pylori*-induced gastritis were predominantly enriched in TGF-β and MAPK signaling pathways. We subsequently quantified the expression levels of TGF-β1 and TAK1 (MAP3K7) in both peripheral blood and gastric mucosa across the study groups. Comparative analysis demonstrated significantly elevated TGF-β1 mRNA expression in both peripheral blood and gastric mucosa of the patients with *H. pylori*-positive gastritis, accompanied by increased TGF-β1 release in peripheral circulation (Figures 4A,B). Immunohistochemical analysis confirmed higher TGF-β1 protein expression in the mucosa of the patients with *H. pylori*-infected gastritis compared to the controls (Figure 4C). Furthermore, the infected group exhibited upregulated TAK1 mRNA and protein expression in the gastric mucosa (Figures 4C,D), along with enhanced TAK1 mRNA levels in peripheral blood (Figure 4D). Notably, phosphorylated TAK1 (p-TAK1) protein expression was markedly increased in the gastric mucosa of the infected patients compared to the controls (Figure 4C). These findings collectively indicate that *H. pylori* infection upregulates TGF-*β*1 and TAK1 expression.

### 3.4 *Helicobacter pylori* infection induces Th17 cell differentiation and increases IL-17 expression

**Figure 5:**
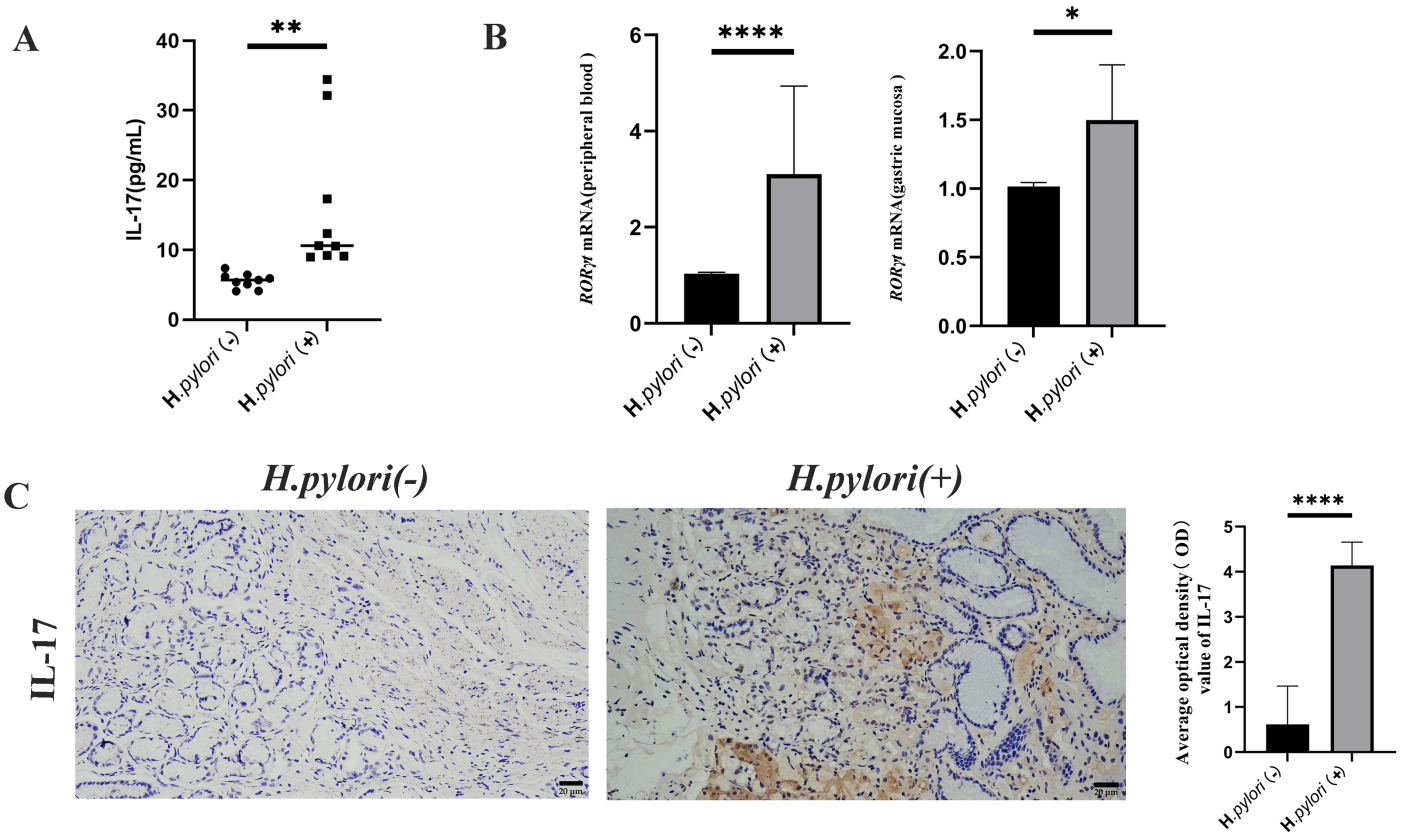
*Helicobacter pylori* colonization increases Th17 cells and IL-17 expression. **(A)** ELISA detection of peripheral blood IL-17 levels in the two groups of patients. **(B)** Real-time quantitative PCR for RORγt mRNA expression in the peripheral blood and gastric mucosa of the patients. **(C)** Expression of IL-17 proteins in the gastric mucosa of the patients by IHC. (IHC, magnification, 200×). *n* ≥ 3, data are presented as the mean ± SD. *P < 0.05, ****P < 0.001, ****p < 0.0001 (Student’s *t* test).

Proteomic analysis revealed that *H. pylori* infection not only induced the activation of the TGF-β signaling pathway but also led to significant enrichment of differentially expressed proteins within the IL-17 signaling pathway. Consequently, we investigated the impact of *H. pylori* infection on Th17 cell expression and associated cytokine secretion. Comparative analysis demonstrated increased IL-17 secretion in peripheral blood (Figure 5A), elevated mRNA expression of the Th17-specific transcription factor RORγt in both peripheral blood and gastric mucosa (Figure 5B), and enhanced IL-17 protein expression in the patients with *H. pylori*-infected gastritis compared to the uninfected controls. Immunohistochemical (IHC) analysis of gastric mucosal paraffin sections confirmed cytoplasmic IL-17 expression in both groups, with significantly higher expression levels observed in the *H. pylori*-positive gastritis group (Figure 5C).

### 3.5 *Helicobacter pylori* infection induces increased Treg cell differentiation and IL-10 expression

**Figure 6:**
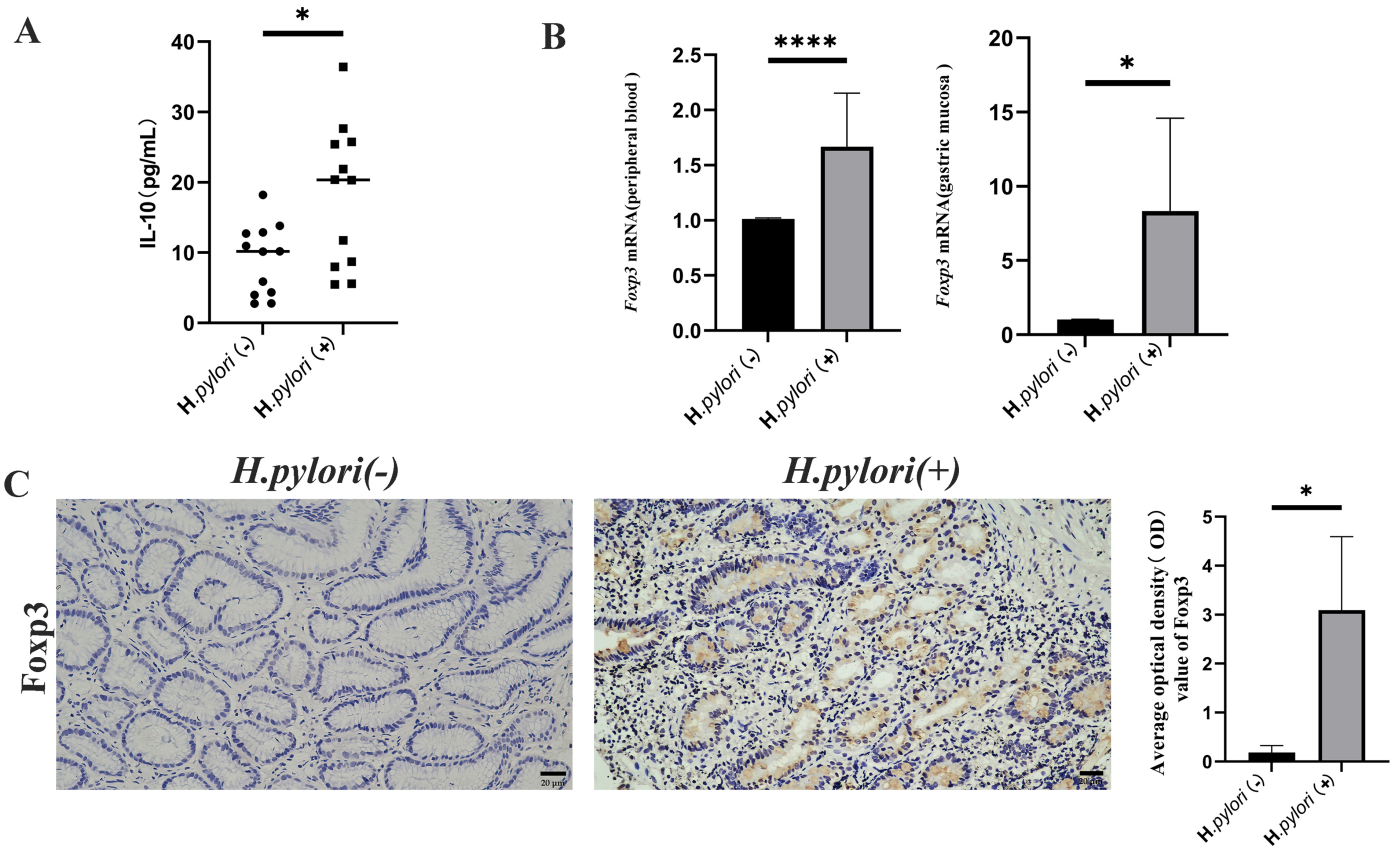
*Helicobacter pylori* colonization increases Treg cell and IL-10 expression. **(A)** ELISA analysis of peripheral blood IL-10 in the two patient groups. **(B)** Real-time quantitative PCR analysis of the Foxp3 mRNA expression in the peripheral blood and gastric mucosa of the patients. **(C)** Expression of Foxp3 proteins in the gastric mucosa of the patients by IHC. (IHC, magnification, 200×). *n* ≥ 3, data are presented as the mean ± SD. *p < 0.05, ****p < 0.001, **** *p* < 0.0001 (Student’s *t-*test).

During infection, Th17 and Treg contribute to the occurrence of inflammation and bacterial colonization in the gastric mucosa, with the Treg cells known to inhibit the immune response of Th17. In this study, we measured the expression levels of Treg and IL-10 using ELISA and found that the peripheral blood levels of IL-10 were significantly higher in the patients with *H. pylori*-infected gastritis compared to the negative group (Figure 6A). The mRNA expression of the Treg cell marker transcription factor Foxp3 was elevated in both peripheral blood and gastric mucosa of the patients infected with *H. pylori* (Figure 6B), and IHC detection of Foxp3 protein revealed that its expression in gastric mucosal tissues was significantly higher in the *H. pylori*-infected gastritis group compared to the *H. pylori*-negative gastritis group (Figure 6C).

### 3.6 *Helicobacter pylori* infection decreases the Th17/Treg ratio in mice with gastritis mice

**Figure 7:**
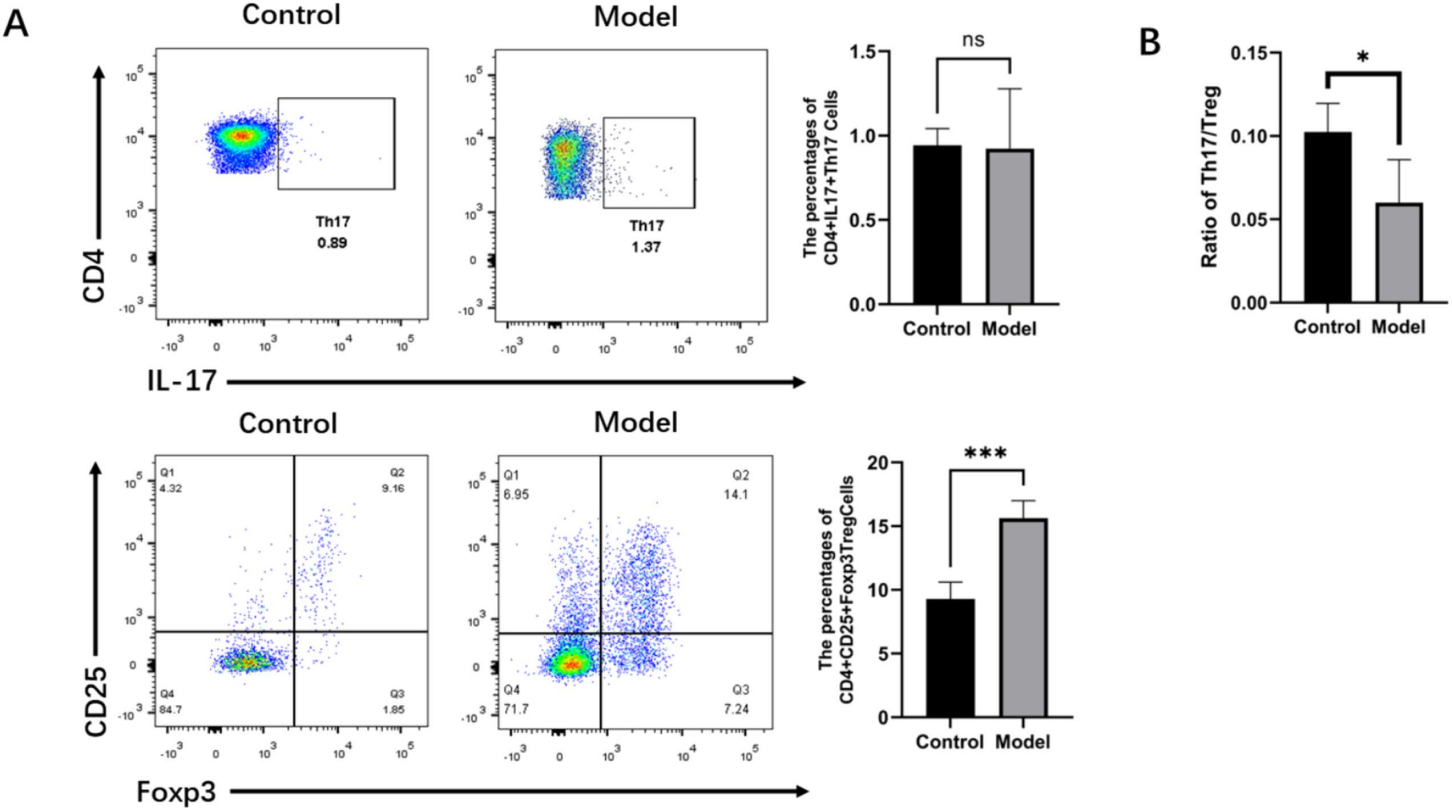
*Helicobacter pylori* infection downregulated the Th17/Treg ratio in the mice with gastritis. **(A)** Representative flow cytometry imaging of the percentage of Tregs and Th17 cells in the spleens of the mice. **(B)** Ratio of Tregs to Th17 cells. *n* ≥ 3, data presented as the mean ± SD. *p < 0.05, ***p < 0.001 (Student’s *t* test).

The elevated expression of RORγt and Foxp3 mRNA in the gastric mucosa and peripheral blood, as well as the increased expression of Th17 and Treg in the gastric mucosa, of the patients with infectious gastritis indicated that Th17 and Treg responses play crucial roles in antimicrobial immunity during *H. pylori* infection. To further determine the relationship between *H. pylori* infection and Th17 and Treg cell differentiation, we analyzed the populations of CD4 + IL-17 + Th17 cells and CD4 + CD25 + Foxp3 + Treg cells in the spleens of mice (Figure 7A). The results showed that there was no significant difference in the CD4 + IL-17 + Th17 cell populations between the model group and the normal group of mice, whereas the number of CD4 + CD25 + Foxp3 + Treg cells in the model group was significantly higher than that in the normal group. Moreover, the Th17/Treg ratio, which indicates immune status, was examined in the *H. pylori-infected* mice group (Figure 7B). It was observed that the Th17/Treg ratio in the mice of the model group was lower compared to the normal group and that *H. pylori* infection polarized CD4 + T cells toward Treg cells, causing a Th17/Treg imbalance. This promoted the Treg cell-mediated immune response, resulting in immune tolerance. This established favorable conditions for the persistent colonization of *H. pylori* and the development of chronic inflammation.

## 4 Discussion

*Helicobacter pylori* (*H. pylori*) exhibits high infectivity and transmissibility, with over half of the global population chronically infected by this pathogen. Utilizing its flagella, the bacterium penetrates the gastric mucus layer and adheres firmly to gastric epithelial cells. Following colonization, the virulence factors of *H. pylori* (e.g., CagA and VacA) trigger extensive differentiation and proliferation of immunocompetent cells while simultaneously inducing the release of both pro-inflammatory and anti-inflammatory cytokines (Yin et al., 2025). This persistent cycle of inflammation and immune evasion not only directly damages the gastric mucosal epithelium but also leads to chronic gastritis, atrophy, and ultimately precancerous lesions (Kalisperati et al., 2017). Consequently, disrupting *H. pylori* colonization and preventing persistent infection are essential strategies to halt the progression of chronic gastritis.

The inflammatory and immune responses during *H. pylori* infection are complex and vary significantly; however, this robust immune reaction is insufficient to eradicate the pathogen and may even exacerbate gastric mucosal inflammation. Research indicates that this inflammatory response may be associated with *H. pylori*-mediated stimulation of Th17 cells and subsequent cytokine release (Brackman et al., 2024). Early studies have demonstrated that regulatory T (Treg) cells also participate in bacterial colonization, chronic inflammatory changes, and the expression of immunosuppressive cytokines (Raghavan and Quiding-Järbrink, 2012; Sehrawat et al., 2015). Both Th17 and Treg cells require shared TGF-*β* signaling for initial differentiation (Wang et al., 2023), and blockade of TGF-*β* signaling has been shown to inhibit Th17 differentiation while improving the Th17/Treg balance during inflammation (Aarts et al., 2022). Given the current lack of consensus regarding the interaction between *H. pylori* infection and Th17/Treg responses, as well as the unclear role of TGF-*β* signaling during *H. pylori* infection, this study employed gastric mucosal proteomic profiling in gastritis patients to identify differentially expressed protein-enriched signaling pathways following *H. pylori* infection for subsequent validation. Our investigation provides a preliminary exploration of the potential role of TGF-*β* signaling in *H. pylori*-induced gastritis. We examine alterations in Th17/Treg cell balance and associated cytokine levels during the infection, thereby offering novel insights that could aid in the prevention and treatment of *H. pylori* infections.

Proteomic analysis of gastric mucosal tissues revealed that differentially expressed proteins during *H. pylori* infection were significantly enriched in the TGF-*β*, MAPK, Th17, and IL-17 signaling pathways. TGF-β may enhance *H. pylori* adhesion and colonization in host cells, potentially contributing to *H. pylori*-induced gastric mucosal inflammation. Previous studies have demonstrated that the *H. pylori* virulence factor VacA suppresses T cell proliferation and immune responses while increasing gastric mucosal adhesion through TGF-*β* upregulation (Li et al., 2015; Altobelli et al., 2019). Our current study identified upregulated expression of key TGF-*β* pathway components (TGF-β1 and USP15) in the gastric mucosa of patients with *H. pylori*-infected gastritis. USP15, a deubiquitinase for TGF-*β* receptors, enhances downstream signaling and promotes RORγt stabilization, thereby facilitating Th17 differentiation. These findings demonstrate that *H. pylori* infection upregulates TGF-β1 expression, playing a pivotal role in gastritis pathogenesis. Furthermore, we observed increased expression of the specific protease USP15 during *H. pylori* infection, which stabilizes TGF-β receptors and their downstream signal transducers, stimulating cell proliferation through enhanced TGF-β activity (Das et al., 2021; Eichhorn et al., 2012). Notably, USP15-mediated RORγt deubiquitination actively promotes Th17 differentiation (He et al., 2016), suggesting close interconnections among TGF-β signaling, Th17 cell differentiation, and *H. pylori* infection.

The MAPK and TGF-*β* signaling pathways play pivotal roles in regulating inflammatory and chemokine expression across various diseases. The non-canonical TGF-β pathway (TAK1-MKK-p38/JNK) amplifies early inflammatory responses, establishing a positive feedback loop between TGF-*β* and MAPK signaling. TGF-β-mediated activation of TAK1 within the MAPK family precisely modulates inflammatory intensity and tissue remodeling, representing a common therapeutic target for multiple pathologies (Chu et al., 2024; Dong et al., 2020; Ren et al., 2020; Zhao et al., 2021). Studies further indicate that the TAK1-regulated balance between anti-apoptotic and inflammatory responses may determine epithelial cell fate during *H. pylori* infection, thereby contributing to gastritis pathogenesis (Hayakawa et al., 2013). Our investigation revealed upregulated TAK1 mRNA expression in both peripheral blood and gastric mucosa of the patients with *H. pylori*-infected gastritis, along with concomitant overexpression of TGF-*β*1 and TAK1 proteins in the gastric mucosa, suggesting *H. pylori*-mediated upregulation of these key TGF-β pathway components. However, the direct causal relationship remains to be validated through TGF-β1/TAK1 inhibition or knockout experiments, which will be addressed in subsequent studies.

TGF-β exhibits pleiotropic functions in Treg and Th17 cell biology, promoting Foxp3 expression to drive Treg cell generation while suppressing Th17 differentiation (Schnell et al., 2021; Wu et al., 2025). However, under Foxp3-deficient conditions, TGF-β paradoxically enhances Th17 cell development (van der Veeken et al., 2022). Following *H. pylori* infection, MHC class II-mediated bacterial antigen presentation to CD4^+^ T cells modulates Th17/Treg differentiation in a TGF-β-dependent manner (Sun et al., 2022). IL-17—a crucial proinflammatory cytokine predominantly secreted by Th17 cells—requires both IL-6 and the transcription factor RORγt for Th17 lineage commitment (Lee et al., 2020). Our study demonstrated upregulated RORγt mRNA expression and increased IL-17 secretion in the patients with *H. pylori*-infected gastritis, which recruit inflammatory cells to the gastric mucosa and mediate host inflammatory responses against the pathogen. Concurrently, we observed elevated Foxp3 mRNA levels in peripheral blood and gastric mucosa, along with enhanced Foxp3 protein expression in mucosal tissues, suggesting that mature Treg recruitment to gastric surfaces may counteract Th17-mediated inflammation. The concomitant increase of TGF-β1 and IL-10 in the patients’ peripheral blood reveals coordinated immunosuppression, with IL-10 exerting anti-inflammatory effects and both cytokines collectively inhibiting naïve T cell activation and restraining excessive Th17 responses. This dynamic interplay between lymphocyte subsets prevents immune hyperactivation while simultaneously creating an immunological niche that facilitates *H. pylori*’s immune evasion and persistent gastric colonization.

Our animal model experiments revealed Th17/Treg imbalance in mice with *H. pylori*-infected gastritis. While Th17 cell populations showed no significant difference between the control and model groups, Treg cells were markedly increased in the infected mice—a finding inconsistent with our clinical observations of elevated Th17 responses in the peripheral blood and gastric mucosa of the *H. pylori*-infected patients. This discrepancy may stem from Foxp3-mediated suppression of Th17 differentiation and function by the highly expressed Treg cells during infection. Notably, emerging research has identified a novel Th17 subset (IL-10-producing Th17) exhibiting remarkable plasticity (Cui et al., 2024). The inherent instability of Th17 cells enables their phenotypic conversion from IL-17-secreting proinflammatory effectors to IL-10-producing anti-inflammatory cells under specific microenvironmental conditions. Consequently, conventional CD4 + IL-17 + detection methods may prove inadequate for evaluating Th17 populations during *H. pylori* infection, as they cannot distinguish these plastic, dual-functional subsets that might preferentially secrete IL-10 in response to bacterial persistence.

In summary, this study provides preliminary evidence of significant upregulation of TGF-β1/TAK1 proteins in the gastric tissues of adult patients with *H. pylori* gastritis, accompanied by concurrent elevation of Th17- and Treg-associated factors (RORγt, Foxp3, IL-17, IL-10) in both peripheral blood and gastric mucosa. In addition, the animal models revealed a decreased Th17/Treg ratio. These findings systematically link, for the first time, the TGF-β1-TAK1 axis with Th17/Treg imbalance during adult *H. pylori* infection, offering novel mechanistic insights. However, since specific inhibition or knockout experiments targeting TGF-β1 or TAK1 have not yet been conducted, the direct causal relationships and precise regulatory mechanisms require further functional validation through subsequent investigations. Such studies will establish an experimental foundation for developing strategies to eradicate *H. pylori* and prevent disease progression.

## Data Availability

The data presented in the study are deposited in the ProteomeXchange Consortium (https://www.proteomexchange.org), accession number PXD068011.
