# Supplementary material for: Gastric mucosal proteomic analysis reveals the role of the TGF-β signaling pathway in gastritis associated with Helicobacter pylori infection
Source: Front Microbiol. 2025 Sep 16;16:1565392. doi: 10.3389/fmicb.2025.1565392 (PMC12479426; doi:10.3389/fmicb.2025.1565392)
Supplement: Supplementary file 2 [file Data_Sheet_2.pdf]

Case \ Test	14C-UBT	RUT	W-S
Wang1	+	−	−
Yang2	+	+	+
Zhang3	−	+	+
Wang4	−	−	−
Liu5	−	+	+
Xu6	−	−	−
An7	none	−	−
Zheng8	+	−	+
Zhang9	−	−	−
Long10	none	+	+
Wu11	+	−	+
.Liu12	−	−	−
Luo13	−	+	−
Wang14	−	−	−
Tan 15	−	−	−
Huang16	+	−	+
Zhang17	+	−	+
Liu18	+	+	+
Xu19	−	none	−
Chu20	+	+	+
Zhang21	+	+	−
Huang22	−	−	−
Shu23	−	−	−
Cheng24	−	+	+
Cheng25	+	+	+
Zhao26	−	−	−
Pan27	+	+	none
Lu28	−	−	−
You29	none	−	−
Xiong30	−	−	−
Liu31	−	−	−
Yang32	+	+	+
Zheng33	−	−	−
Zhou34	+	+	+
Yuan35	−	−	−
Tao36	+	−	+
Mo37	−	−	−
Liu38	−	−	−
Cheng39	+	−	−
Rao40	+	−	+
Wang41	−	−	−

Patient cases were systematically identified and coded using a standardized nomenclature combining surnames with sequential collection numbers

"None" indicates that the patient did not undergo this test

Xu42	−	+	+
Li43	−	−	−
Ling44	+	none	+
Zhu45	−	−	−
Sheng46	−	+	−
Wang47	−	−	−
Hu48	+	+	+
Liu49	−	−	−
Zhou50	+	+	+
Song51	−	−	−
Long52	+	+	−
Zhao53	−	−	−
Yang54	+	−	+
